# The Choice of Operation for Uterine Fibroids

**Published:** 1902-10-18

**Authors:** 


### THE CHOICE OF OPERATION FOR UTERINE FIBROIDS.

In reference to the question discussed in the preceding article, namely, that of the choice of operation in cases of fibroid tumours, the remarks made by Professor Herbert Spencer at Manchester are of interest. He said that when it had been decided that an operation was advisable the question was as to what the operation should be. The ideal operation, if the tumour were pedunculated, was amputation, and if sessile enucleation, either through the cervix or by colpotomy, or by laparotomy. The enucleation of sessile submucous tumours through the cervix gave very good results, provided that the tumour were not larger than an infant's head and were not infected. It was next to the amputation of a polypus, the best operation which was performed for fibroids. But when the tumours were very large, or burrowed deeply, or were multiple, enucleation, whether by the vagina or by the abdomen, was often more dangerous than hysterectomy. For tumours requiring hysterectomy, which were not larger than a fetal head, vaginal hysterectomy was to be preferred, while for large tumours abdominal hysterectomy was the better operation, and of the three forms of abdominal operation among which the choice lay, namely, supravaginal hysterectomy with extraperitoneal treatment of the pedicle, supravaginal hysterectomy with intraperitoneal treatment of the pedicle, and total abdominal hysterectomy, he preferred the latter.

